# Increased Litterfall in Tropical Forests Boosts the Transfer of Soil CO_2_ to the Atmosphere

**DOI:** 10.1371/journal.pone.0001299

**Published:** 2007-12-12

**Authors:** Emma J. Sayer, Jennifer S. Powers, Edmund V. J. Tanner

**Affiliations:** 1 Department of Plant Sciences, University of Cambridge, Cambridge, United Kingdom; 2 Smithsonian Tropical Research Institute, Balboa, Ancon, Panama, Republic of Panama; 3 Department of Ecology, Evolution and Behavior, University of Minnesota, St. Paul, Minnesota, United States of America; 4 Department of Plant Biology, University of Minnesota, St. Paul, Minnesota, United States of America; 5 Department of Soil, Water and Climate, University of Minnesota, St. Paul, Minnesota, United States of America; Centre National de la Recherche Scientifique, France

## Abstract

Aboveground litter production in forests is likely to increase as a consequence of elevated atmospheric carbon dioxide (CO_2_) concentrations, rising temperatures, and shifting rainfall patterns. As litterfall represents a major flux of carbon from vegetation to soil, changes in litter inputs are likely to have wide-reaching consequences for soil carbon dynamics. Such disturbances to the carbon balance may be particularly important in the tropics because tropical forests store almost 30% of the global soil carbon, making them a critical component of the global carbon cycle; nevertheless, the effects of increasing aboveground litter production on belowground carbon dynamics are poorly understood. We used long-term, large-scale monthly litter removal and addition treatments in a lowland tropical forest to assess the consequences of increased litterfall on belowground CO_2_ production. Over the second to the fifth year of treatments, litter addition increased soil respiration more than litter removal decreased it; soil respiration was on average 20% lower in the litter removal and 43% higher in the litter addition treatment compared to the controls but litter addition did not change microbial biomass. We predicted a 9% increase in soil respiration in the litter addition plots, based on the 20% decrease in the litter removal plots and an 11% reduction due to lower fine root biomass in the litter addition plots. The 43% measured increase in soil respiration was therefore 34% higher than predicted and it is possible that this ‘extra’ CO_2_ was a result of priming effects, i.e. stimulation of the decomposition of older soil organic matter by the addition of fresh organic matter. Our results show that increases in aboveground litter production as a result of global change have the potential to cause considerable losses of soil carbon to the atmosphere in tropical forests.

## Introduction

Changes in litter quantity as a consequence of global climate change are becoming increasingly likely; recent FACE experiments have shown that litterfall increases with elevated atmospheric CO_2_ concentrations [1]–[5] and predicted changes in rainfall distribution patterns [6] and temperature [7] may also affect litterfall by altering leafing phenology. As litterfall represents a major pathway for carbon and nutrients between vegetation and soil it seems likely that changes in aboveground litter production will have consequences for belowground processes. However, despite the increasing recognition that research on terrestrial ecosystem dynamics needs a combined aboveground-belowground approach [8], the potential impact of changes in litterfall on belowground carbon dynamics has been largely ignored [9].

Tropical forests are a critical component of the global carbon cycle as they store 20–25% of the global terrestrial carbon [10], [11]. Ongoing debates about whether tropical forests are a source or sink for atmospheric carbon have led to increased interest in the belowground components of their carbon cycle [12] because they also contribute almost 30% to global soil carbon storage [13]. Soil respiration from root and heterotrophic respiration alone releases approximately 80 Pg of carbon into the atmosphere per year to which tropical and subtropical forests contribute more than any other biome [14]. Recent studies have investigated the direct effects of elevated CO_2_ [15], rising temperature [16], [17], and fertilizer [18]–[20] on soil carbon cycling but we know very little about how soil respiration will be affected by the predicted changes in aboveground production caused by global climate change. We believe that an increase in aboveground litterfall may have a large impact on belowground carbon and nutrient cycling, as annual litterfall is closely correlated with soil respiration on a global scale [21], [22], and the amount of litter on the forest floor also affects soil nutrient status, soil water content, soil temperature, and pH [9], all of which can influence soil respiration rates. To investigate this, we conducted an experiment consisting of large-scale monthly litter removal (L-) and litter addition (L+) treatments in a lowland tropical forest. Our results show that an increase in aboveground litterfall caused a disproportionate increase in soil respiration, reduced the amount of carbon allocated to fine root biomass and thus has the potential to cause substantial losses of carbon belowground.

## Results

### Soil respiration

**Figure 1:**
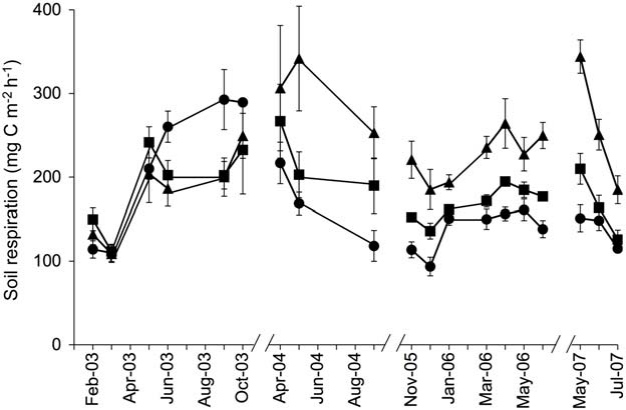
Soil respiration from February 2003 to July 2007 in litter manipulation treatments in tropical rainforest. Soil respiration rates were measured over bare soil in all treatments; squares are controls, triangles are litter addition treatments, and circles are litter removal treatments; error bars show standard errors of means for *n* = 5.

Soil respiration showed a seasonal pattern with low rates (*c*. 130 mg C m^−2^h^−1^) in the dry season and much higher rates (*c.* 260 mg C m^−2^h^−1^) in the wet season. There was no effect of litter manipulation during 2003, the first year of treatments, but from May 2004 (17 months after litter manipulation commenced), respiration from the mineral soil in the litter removal (L-) plots was on average 20% lower than in the control (CT) plots (Fig. 1).

**Figure 2:**
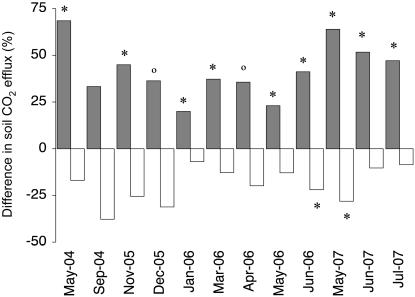
Differences in soil respiration between litter manipulation treatments and controls in tropical rainforest. The differences are calculated as a percentage of the average respiration measured in the control plots for each month; grey bars are litter addition plots and white bars are litter removal plots; a star above a bar denotes a significant treatment effect (P<0.05) compared to the controls, a circle above a bar denotes a marginally significant treatment effect (P<0.065) compared to the controls.

From the second year of treatments, litter addition increased respiration from the mineral soil more than litter removal decreased it. On average, soil respiration in the litter addition (L+) plots was 43% higher than in the controls and the increase was significant or marginally significant in eleven out of twelve months while respiration in the L- plots was significantly lower than the CT plots in only two out of twelve months (Fig. 2). The smallest increase in the L+ plots relative to the CT plots was during the dry season (20% in January 2006; Fig. 2), while the greatest increases were observed during the dry-to-rainy season transition (69% in May 2004 and 64% in May 2007; Fig. 2). The strong increase in soil respiration in the L+ plots was sustained until the end of the study in July 2007 (Fig. 1).

### Soil temperature and soil water content

Soil temperature (0–100 mm depth) varied very little throughout the year; litter removal decreased soil temperature by *c.* 0.5°C relative to the CT and L+ plots during the rainy season only in 2003 and 2004 (P = 0.002); soil temperature did not differ between the L+ and CT plots except in June and July 2007, when it was 0.3°C (P = 0.019) and 0.4°C higher (P = 0.002), respectively, in the L+ plots. Soil water content from 0–60 mm depth was not affected by litter manipulation in any season or year.

### Fine root and microbial biomass

**Figure 3:**
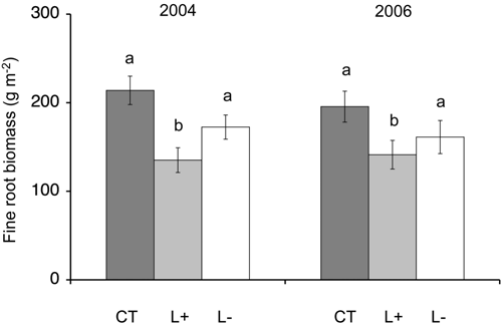
Fine root biomass in the mineral soil (0–100 mm) in litter manipulation treatments in tropical rainforest. CT is control, L+ is litter addition, L– is litter removal; error bars show standard errors of means for *n* = 5; different letters above bars indicate a significant difference between treatments at P<0.05. Data for 2004 has been previously published in a different form [25].

Fine root biomass in the mineral soil (0–100 mm depth) was 37% lower in the L+ plots than in the CT plots in June and July 2004, after 19 months of litter addition and removal treatments (P<0.01; Fig. 3) and 28% lower in August 2006, after 41 months (P = 0.05; Fig. 3). There was no significant difference in fine root biomass between CT and L- plots in either year.

**Figure 4:**
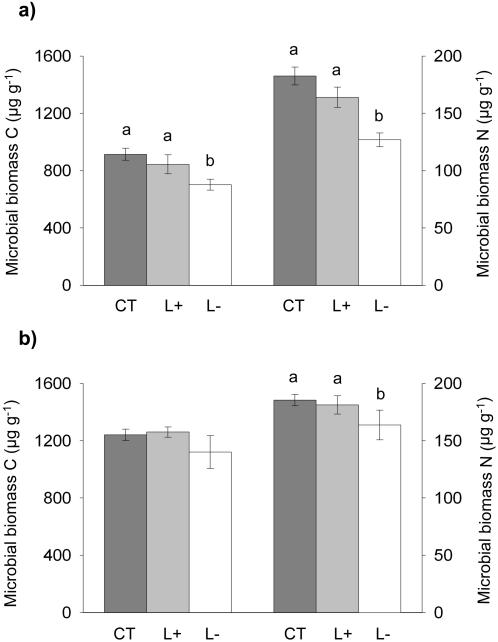
Microbial biomass in the mineral soil (0–100 mm) in litter manipulation treatments in tropical rainforest. Data are given as microbial carbon and nitrogen in a) August 2004, and b) June 2006; CT is control, L+ is litter addition, L– is litter removal; error bars show standard errors of means for *n* = 5; different letters above bars indicate a significant difference between treatments at P<0.05.

Total microbial C and N (0–100 mm depth) had decreased by 23% in the L- plots relative to the control treatment in August 2004 (P = 0.011 and P = 0.003 for C and N, respectively; Fig. 4a) and microbial N in the L- plots was 18% lower than in the CT plots in June 2006 (P = 0.006; Fig. 4b).

Litter addition had no significant effect on microbial biomass C and N in either year.

## Discussion

We attribute the lack of significant responses in soil respiration to the experimental treatments during the first year to a combination of two factors: firstly we started treatments during the dry season when decomposition is limited by the lack of moisture [23] and secondly, we did not include the litter layer in our measurements of soil respiration. Thus, we would not expect CO_2_ efflux from the mineral soil to be affected by our treatments until decomposition processes were sufficiently advanced to affect the input of carbon and nutrients to the mineral soil.

The 20% reduction in soil respiration observed in the litter removal treatment from the second year of treatments until the end of the study is similar to the 28% decrease reported in plots in young regrowth forest in Brazil after one year of litter removal, where controls included the litter layer in CO_2_ efflux measurements [24], but lower than the 51% decrease after seven years of litter removal in lower montane forest in Puerto Rico [25]. We can attribute the decrease in our study principally to a reduction in heterotrophic respiration due to the withdrawal of fresh substrate, as there were no differences in fine root biomass in the upper 100 mm of the mineral soil between CT and L- plots in 2004 or in 2006 (Fig. 3). Furthermore, we found no significant differences in soil water content between treatments and the small (≤0.5°C) and inconsistent differences in soil temperature were unlikely to affect soil respiration.

**Figure 5:**
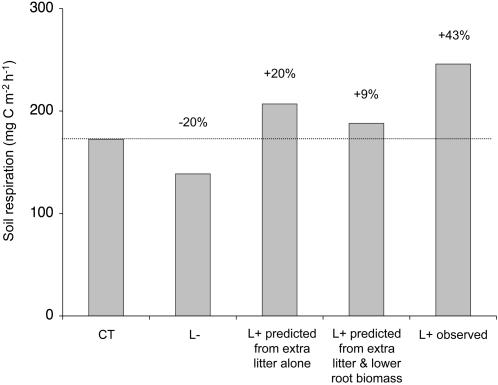
Comparison of predicted and observed soil respiration in litter manipulation plots in tropical rainforest. Differences are expressed as percentages of the mean rate measured in controls from May 2004 to July 2007; CT is control, L+ is litter addition, L– is litter removal.

We expected an average increase of 20% in soil respiration in the L+ plots during the period from May 2004 to July 2007, as CO_2_ efflux from the mineral soil decreased by this percentage in the L- plots. However, soil respiration in the L+ plots was on average 43% higher than the controls and therefore 23% higher than expected by the addition of litter alone. Furthermore, this increase was sustained from May 2004 until the end of the study in July 2007 (Fig. 2). The increase in soil respiration in the L+ plots is considerable and greater than the effects of fertilization with 150 kg ha^−1^ yr^−1^ of phosphorus in a study in Costa Rica [20]. While fertilization treatments are thought to boost soil respiration by removing the nutrient limitation of decomposition processes [20], [26], and increasing microbial biomass [26], we found no increase in leaf litter decomposition rates in our L+ treatments [27], and no changes in microbial biomass C or N (Fig. 4a,b). Furthermore, litter addition decreased fine root biomass in the mineral soil by 37% in 2004 [28] and 30% in 2006 (Fig. 3). Fine roots contribute the bulk of root respiration [29]–[30], root respiration is proportional to root biomass [31], and it typically makes up at least 30% of total soil respiration in the tropics [31]–[34]; consequently the lower fine root biomass in the L+ plots would effectively reduce soil respiration by *c.* 11%. The expected increase in soil respiration in the L+ plots due to litter addition and reduced fine root biomass would therefore only be *c*. 9% relative to the controls. Thus, measured soil respiration was 34% higher than expected from the extra litter and reduced root biomass (Fig. 5). This suggests that litter addition, besides increasing the amount of readily degradable carbon, may also cause substantial losses of CO_2_ from the soil. It is likely that this extra CO_2_ production is attributable to priming effects, the enhanced microbial decomposition of older, more recalcitrant soil organic matter by the addition of fresh organic matter [35], [36]. Strong pulses of soil respiration have previously been observed in lowland tropical rainforest during the dry-to-rainy season transition and were interpreted as a ‘natural priming effect’ caused by large amounts of water-soluble carbon leaching from the litter that had accumulated during the dry season [20]. Our study shows that additional leaf litter has the potential to sustain priming effects throughout the year.

Estimated annual soil respiration rates were 15.3 t C ha^−1^ yr^−1^, 19.0 t C ha^−1^ yr^−1^, and 13.7 t C ha^−1^ yr^−1^ for the CT, L+, and L- treatments, respectively. Thus, the soil carbon lost to the atmosphere in our litter addition treatment is at least 4.4 t C ha^−1^ yr^−1^and may be as high as 6.5 t C ha^−1^ yr^−1^ (23% and 34%, respectively, of expected soil respiration in the L+ plots). Laboratory incubations have demonstrated that repeated additions of fresh organic matter to soil induce greater priming effects than a single addition [37], [38] and that increased decomposition of soil organic matter continued even when the added fresh organic matter had been completely depleted [39]. We therefore expect that chronic increases in litterfall will induce a substantial release of soil carbon in the medium term.

Thus, we show for the first time that increased aboveground litter production in response to global climate change may trigger priming effects and convert considerable amounts of soil carbon to atmospheric carbon dioxide.

## Materials and Methods

### Site description

The study was carried out as part of an ongoing long-term litter manipulation experiment to investigate the importance of litterfall in the carbon dynamics and nutrient cycling of tropical forests. The forest under study is an old-growth moist lowland tropical forest, located on the Gigante Peninsula (9°06′N, 79°54′W) of the Barro Colorado Nature Monument in Panama, Central America. The soil is an oxisol with a pH of 4.5–5.0, with low ‘available’ phosphorus concentration, but high base saturation and cation exchange capacity [28; 40]–[41]. Nearby Barro Colorado Island (*c*. 5 km from the study site) receives a mean annual rainfall of 2600 mm and has an average temperature of 27°C [42]. There is a strong dry season from January to April with a median rainfall of less than 100 mm per month [43]; almost 90% of the annual precipitation occurs during the rainy season. Fifteen 45-m×45-m plots were established within a 40-ha area (500-m×800-m) of old-growth forest in 2000. In 2001 all 15 plots were trenched to a depth of 0.5 m in order to minimize lateral nutrient- and water movement via the root/mycorrhizal network; the trenches were double-lined with plastic and backfilled. Starting in January 2003, the litter (including branches ≤100 mm in diameter) in five plots was raked up once a month, resulting in low, but not entirely absent, litter standing crop (L- plots). The removed litter was immediately spread on five further plots, approximately doubling the monthly litterfall (L+ plots); five plots were left as controls (CT plots). The assignment of treatments was made on a stratified random basis, stratified by total litterfall per plot in 2001, i.e. the three plots with highest litterfall were randomly assigned to treatments, then the next three and so on.

### Soil respiration

In October 2002 four measurement sites were established in each of the 15 plots; collars made of PVC pipe of 108 mm inner diameter and 44 mm depth were placed 12.5 m into the plot, measured from the centres of each of the fours sides of the plot. The collars were sunk into the soil to 10 mm depth and anchored using small plastic tent pegs, which were attached to the collars by cable binders and sunk diagonally into the ground to avoid channelling water into the soil under the collars. The collars were left undisturbed throughout the experiment. Soil respiration from the mineral soil was measured in the collars in February, March, May, June, September and October of 2003, and April, May, and September of 2004 using an infra-red gas analyser (IRGA) Li-6400 with an Li-6400-9 soil chamber attachment (LI-COR, Lincoln, USA). The ambient CO_2_ level was determined for each site individually and measurements started at 5 ppm below the ambient CO_2_ level. Three measurements were taken over each collar at each time and the values were averaged to give one value per collar per time.

In September 2005 new measurement sites were set up adjacent to those established in 2002. PVC collars of 200 mm diameter and 120 mm depth were sunk into the ground to 20 mm and anchored with tent pegs as described above. The collars were set up two months before starting measurements and were left undisturbed throughout the experiment. Soil respiration was measured over the new collars in November and December 2005, January, March, April, May, and June 2006, and May, June and July 2007 using the Li-8100 soil CO_2_ flux system (LI-COR, Lincoln, USA). The ambient CO_2_ level was determined for each site automatically and one measurement of 2 minutes duration was taken over each collar. As our main research aim was to determine whether the amount of litter on the forest floor affects respiration from the mineral soil, leaf litter was removed from the collars prior to all measurements; great care was taken not to disturb the underlying mineral soil, and the litter was replaced once the measurements had been completed.

All measurements were made during 1–3 days each month between 8.00 h and 14.00 h. If measurements could not be completed in one day, they were made over consecutive days whenever possible, but no measurements were taken during or immediately following heavy rainfall. When measurements were taken over several days, an equal number of plots per treatment was measured each day; the plot means (four collars per plot) were used for statistical analysis of treatment effects.

Annual respiration rates were estimated by obtaining a daily mean each for the rainy and dry seasons from the data collected, multiplying the daily mean by the average number of days in each season (135 days for the dry season, 230 days for the rainy season), and then summing the obtained values.

### Soil temperature and soil water content

Soil temperature was recorded during respiration measurements in 2003, 2004, 2006, and 2007 within 0.5 m of the collars using the IRGA's integrated soil temperature probe inserted to a depth of 100 mm. Volumetric soil water content was measured from 0–60 mm depth using a thetaprobe (Delta-T Devices, Cambridge, UK), which was calibrated to the soil type in the plots following the procedure described by Delta-T. Due to technical problems, volumetric soil water measurements were not made in 2004 or 2005 and only in April in 2006. Gravimetric soil water content was determined in May, June, and August 2004, and in January and June 2006 from four 20-mm diameter soil cores per plot, taken from 0–100 mm depth; volumetric soil water content was then calculated from the gravimetric measurements.

### Fine root and microbial biomass

The biomass of fine roots (≤2 mm diameter) from 0–100 mm depth in the mineral soil was determined in June and July 2004 from ten randomly located 51-mm diameter soil cores per plot [25], and in June 2006 from seven randomly located soil cores per plot; live and recently dead fine roots were carefully separated from the soil by washing in a 0.5-mm mesh sieve and then dried to constant weight in the oven at 70°C.

Total microbial biomass of the mineral soil was measured in August 2004 and June 2006 (during the rainy season). Four soil cores were taken from 0–100 mm depth at the four corners of the inner 20-m×20-m in each plot using a 20-mm diameter punch-corer; the cores were bulked to give one sample per plot. Subsamples were taken to determine soil gravimetric water content and total microbial biomass was determined by the fumigation extraction method [44]–[45]. Briefly, pairs of unfumigated and chloroform-fumigated (exposed to chloroform for three days in the dark) soil samples were shaken in 0.5 M K_2_SO_4_ for one hour, filtered through Whatman No. 1 filter paper, and frozen until analyzed. Total organic carbon (TOC) and total nitrogen in the extracts were measured simultaneously on a TOC VCPH/CPN Total Organic Carbon and Nitrogen Analyzer (Schimadzu, Kyoto, Japan). Total microbial carbon and nitrogen were estimated as the difference between fumigated and unfumigated samples (expressed on an oven-dry mass basis), divided by appropriate conversion factors [44]–[45].

### Statistical analyses

Using mean values per plot (i.e *n* = 5 for each treatment and control) differences among treatments in soil respiration rates, soil temperature, and soil moisture were investigated by separate repeated measures ANOVAs for each year. Fine root biomass and microbial biomass C and N were analysed with one-way ANOVAs for each year separately. Where treatment effects were found to be significant or marginally significant (P<0.07), post-hoc comparisons were made using Fisher's LSD test. All analyses were carried out in Genstat 7.2 (VSN International Ltd., Hemel Hempstead, UK).
